# Prospective longitudinal study on fear of cancer recurrence in patients newly diagnosed with head and neck cancer: Course, trajectories, and associated factors

**DOI:** 10.1002/hed.26985

**Published:** 2022-01-27

**Authors:** Esther Deuning‐Smit, José A. E. Custers, Špela Miroševič, Robert P. Takes, Femke Jansen, Johannes A. Langendijk, Chris H. J. Terhaard, Robert J. Baatenburg de Jong, C. René Leemans, Johannes H. Smit, Linda Kwakkenbos, Irma M. Verdonck‐de Leeuw, Judith B. Prins

**Affiliations:** ^1^ Department of Medical Psychology Radboud Institute for Health Sciences, Radboud University Medical Center Nijmegen The Netherlands; ^2^ Department of Family Medicine Faculty of Medicine, University of Ljubljana Ljubljana Slovenia; ^3^ Department of Otorhinolaryngology—Head and Neck Surgery Radboud University Medical Center Nijmegen The Netherlands; ^4^ Department of Otolaryngology—Head and Neck Surgery Cancer Center Amsterdam, Amsterdam UMC, Vrije Universiteit Amsterdam Amsterdam The Netherlands; ^5^ Department of Clinical, Neuro and Development Psychology Amsterdam Public Health Research Institute, Vrije Universiteit Amsterdam Amsterdam The Netherlands; ^6^ Department of Radiation Oncology University Medical Center Groningen, University of Groningen Groningen The Netherlands; ^7^ Department of Radiotherapy University Medical Center Utrecht The Netherlands; ^8^ Department of Otolaryngology and Head and Neck Surgery Erasmus Cancer Institute, ErasmusMC Rotterdam The Netherlands; ^9^ Department of Psychiatry Amsterdam Public Health Research Institute, Amsterdam UMC, Vrije Universiteit Amsterdam Amsterdam The Netherlands; ^10^ Clinical Psychology, Behavioural Science Institute Radboud University Nijmegen The Netherlands

**Keywords:** coping, fear of cancer recurrence, head and neck cancer, latent class growth analysis, trajectories

## Abstract

**Background:**

This study assessed the course of fear of cancer recurrence (FCR) in patients newly diagnosed with head and neck cancer (HNC), identified FCR trajectories and factors associated with FCR trajectories.

**Methods:**

Six hundred and seventeen HNC patients from the NET‐QUBIC cohort study completed the Cancer Worry Scale‐6 at diagnosis, 3 and 6 months post‐treatment. FCR trajectories were identified using Latent Class Growth Analysis. Associations were explored between FCR trajectories and baseline demographic and medical variables, coping and self‐efficacy.

**Results:**

Overall, FCR decreased slightly between baseline and 3 months post‐treatment and remained stable up to 6 months. Two FCR trajectories were identified: “high stable” (*n* = 125) and “low declining” (*n* = 492). Patients with high stable FCR were younger, reported more negative adjustment, passive coping, and reassuring thoughts, and less avoidance.

**Conclusions:**

The majority of HNC patients have low declining FCR after diagnosis, but one in five patients experience persistent high FCR up to 6 months post‐treatment.

## 1 INTRODUCTION

Fear of cancer recurrence (FCR) is “*the fear, worry or concern that cancer will return or progress*”.^1^ It is one of the most important concerns^2^, ^3^, ^4^ and unmet needs for help^2^, ^5^, ^6^ amongst patients with head and neck cancer (HNC). Across disease phases, 31% to 83% of HNC patients report mild to high levels of FCR.^7^, ^8^, ^9^, ^10^, ^11^, ^12^ While some fear is assumed adaptive, as it enables adequate self‐monitoring of symptoms and positive health behavior,^13^, ^14^, ^15^ high FCR interferes with daily and social functioning and is associated with reduced quality of life (QoL)^9^, ^11^, ^13^, ^16^ and increased health care use.^17^ Persistent high FCR is regarded clinically relevant, and these patients may benefit from psychological treatment.^18^ However, limited data exist on how FCR develops after HNC diagnosis and which subgroups of patients are at risk for persistent high FCR.

Four longitudinal studies of FCR in HNC patients that were published to date, with follow‐up periods between 7 months and 2 years^10^ and using between two^8^, ^10^ and six^7^ assessments, suggested that high FCR is stable over time.^7^, ^8^, ^9^, ^10^ However, these studies were limited by small samples, heterogeneous definitions and measurement of (high) FCR, and variable timing of assessments. Furthermore, FCR was assessed at group level. While mean FCR scores and prevalence rates appear stable, FCR likely fluctuates within (subgroups of) patients. In other cancer types, subgroups have been identified with distinct courses of FCR.^19^, ^20^, ^21^, ^22^, ^23^ In HNC patients, descriptive data suggest that some patients experience persistent high or low FCR, while others show fluctuating levels,^9^ but the existence of distinct FCR trajectories has not been assessed.

While HNC recurrence rates are high, most studies found no association between FCR and objective indices of severity (e.g., TNM stage).^8^, ^9^, ^13^, ^16^, ^24^ Instead, several patient‐related factors were associated with higher FCR, including younger age, female sex, smoking and psychological factors including optimism, neuroticism, social support, coping, and self‐efficacy.^12^, ^13^, ^16^, ^24^, ^25^ Although coping plays a central role in theoretic models of FCR,^26^ little is known about its relationship with high FCR in HNC. Coping is described as a dynamic process of cognitive and behavioral effort to manage specific external or internal demands that are perceived as exceeding the person's resources.^27^ Dysfunctional coping has been suggested to play a key role in the persistence of FCR over time by increasing the vulnerability for FCR triggers and reinforcing dysfunctional beliefs regarding recurrence.^15^, ^28^ Previous cross‐sectional^29^, ^30^, ^31^, ^32^, ^33^, ^34^ and longitudinal^23^, ^29^, ^30^ studies in other cancers showed that higher FCR was associated with more avoidance/denial coping, problem‐solving and reassurance seeking.

Self‐efficacy is defined as “people's beliefs about their capabilities to exercise control over events that affect their lives”.^35^ Conceptually, lower self‐efficacy contributes to an increased sense of vulnerability to illness,^36^ which is argued to be characteristic for FCR.^37^ Furthermore, higher self‐efficacy is thought to contribute to adaptive coping.^35^ Studies in breast, prostate and testicular cancer found an association between self‐efficacy and FCR.^23^, ^34^, ^38^, ^39^, ^40^ To our knowledge, self‐efficacy has not yet been examined in relation to FCR in HNC.

This study aims to assess (1) the course of FCR from diagnosis to 6 months post‐treatment, (2) FCR trajectories, and (3) associations of FCR trajectories with baseline demographic and medical variables, coping and self‐efficacy. Understanding the course of FCR and associated factors may contribute to early identification of patients who might benefit from evidence‐based FCR interventions.^41^, ^42^

## 2 PATIENTS AND METHODS

### 2.1 Patients and procedure

This study used data from an ongoing cohort study investigating the course of QoL in HNC patients and their caregivers^43^ (NET‐QUBIC study; www.kubusproject.nl). Data from 739 newly diagnosed HNC patients were collected at baseline (before treatment started), 3 and 6 months post‐treatment. Post‐treatment was defined as the time since the treatment end date, and in case of multiple treatments, the end date of the last treatment. Only patients with complete data for the Cancer Worry Scale‐6 (CWS‐6) on at least one time point were included in the analyses.

Patients were recruited between March 2014 and June 2018 in five head and neck oncological centers and three collaborating general hospitals in the Netherlands. All newly diagnosed HNC patients were screened for eligibility by their treating physician. Eligible patients were: (1) at least 18 years of age; (2) newly diagnosed with HNC (larynx, hypopharynx, oropharynx, oral cavity, unknown primary; all stages); (3) previously untreated and currently planned treatment with curative intent according to standard treatment guidelines; and (4) proficient in the Dutch language. Exclusion criteria were (1) malignancies of the salivary glands, nasopharynx or skin, lymphoma, thyroid cancer and (2) severe psychiatric comorbidities (e.g., schizophrenia, Korsakoff's syndrome, and severe dementia). Eligible patients were invited to participate by their treating physician and received written information by the research nurse or researcher, after which written informed consent was obtained. Self‐report questionnaires were sent by mail and filled out on paper.

Ethical approval was obtained by the coordinating center (Institutional Review Board of Amsterdam UMC2013.301(A2018.307)‐NL45051.029.13) and all participating centers. A detailed description of the procedure and recruitment has been published.^43^, ^44^

### 2.2 Measures

*Fear of cancer recurrence* was assessed using the CWS‐6,^45^ which includes six items that are rated on a four‐point Likert scale. Total scores range from 6 to 24, with higher scores indicating more worry. The CWS‐6 is reliable and has been validated in a Dutch sample of cancer survivors.^46^ CWS‐6 scores ≥12 represent high FCR.^46^ The reliability in this sample was good (Cronbach's *α* = 0.89).

*General coping reactions* were measured using the Summary Positive Adjustment and Summary Negative Adjustment subscales^47^ of the Mental Adjustment to Cancer scale (MAC).^48^ This disease‐specific scale includes 40 items rated on a four‐point Likert scale.^48^ The Dutch version of the MAC showed acceptable psychometric properties of the Summary Scales in a mixed sample of patients with cancer.^49^ The reliability in this sample was good (Cronbach's *α* = 0.76 and 0.85, respectively).

*Specific coping strategies* were assessed with the Utrecht Coping List (UCL) which consists of 47 items rated on a four‐point Likert scale.^50^ The seven subscales include active coping, passive coping, avoiding, palliative coping, seeking social support, expressing emotions and reassuring thoughts. Higher scores indicate more of the coping strategy used. Psychometric properties in a sample of healthy participants were good.^51^ The reliability in our sample was acceptable (Cronbach's *α* = 0.63–0.86).

*Self‐efficacy* was assessed with the 10‐item General Self‐Efficacy scale (GSE) measuring beliefs regarding one's ability to control one's environment and life circumstances.^52^ Items are scored on a four‐point Likert scale. Total scores range from 10 to 40 with higher scores indicating greater self‐efficacy. The reliability in this sample was good (Cronbach's *α* = 0.91).

*Clinical and demographic characteristics* were collected via self‐report questionnaires and medical records, including age, sex, living arrangement, educational status, tumor location, clinical TNM stage (I, II, III, IV), treatment modality, Human papillomavirus (HPV) status and WHO performance status (0—normal activity; 1—restricted in physical activity, ambulatory, light work; 2—ambulatory, capable of all self‐care, unable in any work; 3—only limited self‐care). Physical comorbidity was assessed with the 27‐item Adult Comorbidity Evaluation‐27 index (ACE‐27), which measures presence and severity of individual medical illnesses, graded in four categories (none, mild, moderate, or severe).^53^

### 2.3 Statistical analysis

Data were analyzed using SPSS version 25. Missing items on the CWS‐6 resulted in a missing total score. Missing items on the MAC and UCL were replaced by the participants' subscale mean if at least 80% of the items were answered. Differences in demographic and clinical variables were explored between responders and non‐responders (patients missing CWS‐6 scores on all timepoints) using *t*‐tests and chi‐square tests. Furthermore, differences were explored between completers and non‐completers (patients missing at least one CWS‐6 total score).

Descriptive statistics were used to characterize the sample (means and standard deviations [SD] for continuous variables and percentages for categorical variables). The percentage of participants scoring above the threshold for high FCR (CWS‐6 ≥ 12) was determined for each assessment. The course of FCR over time (CWS‐6 scores) was analyzed for completers using repeated measures ANOVA. Post‐hoc tests examined changes between specific timepoints.

Trajectories (classes) of FCR over time were identified with Latent Class Growth Analysis (LCGA) with maximum likelihood estimation using MPlus version 8.3 following the guidelines of Jung and Wickrama.^54^ This analysis included completers and non‐completers. The optimal number of classes was selected based on fit indices, model parsimony, and usefulness. Criteria for the model fitting the data best were the smallest Bayesian Information Criterion (BIC), significant *p*‐values (*p* < 0.05) for the Bootstrap likelihood ratio Test (BLRT) and the Lo–Mendell–Rubin likelihood ratio test (LMR‐LRT) a higher entropy statistic (approaching 1), and higher posterior probabilities of group membership (approaching 1). The usefulness of the trajectories was evaluated based on the group size (> 5% of the total sample size), intercept and slope. After selecting the most optimal model, patients were assigned to a class based on the most likely group membership.

The association of FCR trajectory with a priori defined baseline demographic variables (age, sex), medical variables (cancer site, cancer stage, HPV status, comorbidity), coping, and self‐efficacy was assessed using chi‐square tests for categorical variables and ANOVA's for continuous variables. Variables significantly related to FCR trajectories (*p* < 0.05) were entered in two separate logistic regression analyses for UCL and MAC to prevent shared variance by two scales measuring similar constructs (coping). The assumption of linearity was tested by adding the interaction term between the predictors and their log transformations in the model. Multicollinearity was assessed using inter‐predictor correlations, tolerance (> 0.01) and VIF (< 10) statistics.

## 3 RESULTS

### 3.1 Patients characteristics

Of the 739 patients in NET‐QUBIC, 617 had CWS‐6 data and were analyzed (122 non‐responders). At baseline, 559 patients completed the CWS‐6; 538 and 485 patients at, respectively, 3 and 6 months post‐treatment. Of 617 participants, 417 (68%) completed the CWS‐6 on all timepoints. Demographic and medical characteristics are presented in Table 1.

**TABLE 1:** Sociodemographic and clinical characteristics (N = 617)

Characteristics	Mean	SD
*Age, years*	63.5	9.4
*Number of treatments received* ^a^	1.5	0.56
	**No. of patients**	**%**
*Sex*		
Men	457	74.1
Women	160	25.9
*Living arrangement* ^1^		
Alone	126	22.1
Cohabiting	445	77.9
*Educational status* ^2^		
Primary education	28	4.9
Lower or preparatory vocational education	114	20
Intermediary general secondary education	94	16.5
Senior general secondary education	107	18.8
Higher general secondary education	44	7.7
Higher professional education	121	21.2
University	62	10.9
*Cancer site*		
Oral cavity	174	28.2
Oropharynx	216	35
Hypopharynx	39	6.3
Larynx	169	27.4
Unknown primary	19	3.1
*Clinical disease stage*		
0^b^	1	0.2
I	149	24.1
II	112	18.2
III	101	16.4
IV	254	41.2
*HPV status (applicable only in oropharyngeal cancer* ^3^ *)*		
Positive	112	59.9
Negative	75	40.1
*Treatment—surgery*		
Yes	202	32.7
No	415	67.3
*Treatment—radiotherapy*		
Yes	485	78.6
No	132	21.4
*Treatment—chemotherapy*		
Yes	190	30.8
No	427	69.2
*Treatment—CO*_*2*_ *‐laser (applicable only in oral cavity and larynx cancer)*		
Yes	46	13.4
No	297	86.6
*WHO*		
Normal activity (0)	435	70.5
Restricted in physical activity, ambulatory, light work (1)	156	25.3
Ambulatory, capable of all self‐care, unable in any work (2)	25	4.1
Only limited self‐care (3)	1	0.2
*Comorbidity* ^4^		
None	184	31.2
Mild	223	37.9
Moderate	121	20.5
Severe	61	10.4

*Note*: Due to missing data: ^1^ *n* = 571, ^2^ *n* = 570, ^3^ *n* = 187, ^4^ *n* = 589.

Abbreviation: HPV, Human papillomavirus.

^a^ All patients received at least one treatment.

^b^ pTNM was Stage 2.

Responders (*N* = 617), compared with non‐responders (*N* = 122) had relatively more often stages I and II disease (24% vs. 11% and 18% vs. 16%), and less stages III and IV disease (16% vs. 21% and 41% vs. 52%, respectively). Furthermore, responders had more often a positive HPV status (60% vs. 43%) and more often none or mild comorbidity (31% vs. 18% and 38% vs. 37%). There were no significant differences regarding age, sex, tumor site, and treatment (Table S1). Completers (*N* = 417) compared to non‐completers (*N* = 200) had relatively more often a positive HPV status (66% vs. 49%) and more often none or mild comorbidity (34% vs. 25% and 41% vs. 32%). There were no significant differences regarding age, sex, tumor site disease stage and treatment (Table S1).

### 3.2 Course of FCR

At baseline, 30.8% of participants scored above the cut‐off indicating high FCR. At 3 and 6 months post‐treatment, this was 24.3% and 20.2%, respectively. Results of the ANOVA analysis (completers, *N* = 417) showed a statistically significant effect of time on FCR (*F*(1.80, 747.46) = 12.48, *p* < 0.001, *η* ^*2*^ = 0.029; Greenhouse–Geisser correction). Post‐hoc tests using Bonferroni correction revealed that mean CWS‐6 scores were significantly higher on baseline (*M* = 10.07, SE = 0.16) compared to 3‐months (*M* = 9.51, SE = 0.15, *p* = 0.001) and 6 months follow‐up (*M* = 9.36 SE = 0.16, *p < 0*.001). Differences in CWS‐6 scores between 3 months and 6‐months were not significant (*p* = 0.632).

### 3.3 Identification of FCR classes

Using LCGA for the total sample (*N* = 617), a two‐class model was identified as most appropriate (Table 2). A four‐class model was not optimal based on all fit indices except for the BLRT test, and one group with *n* < 31 (5% of the total sample size). In the two‐class model, compared with the three‐class model, the entropy and posterior probabilities were better but the BIC was higher. In the three‐class model, a subgroup was identified including 30 patients with a relatively high intercept and steep positive slope. Although this group could reflect a clinically relevant subgroup of patients with high increasing FCR levels, the small size and wide confidence intervals of slope and intercept, together with other fit indices favored a two‐class model.

**TABLE 2:** Fit indices, entropy, and average posterior probabilities across models with different number of classes representing trajectories of fear of cancer recurrence

No. of classes	BIC	LMR‐LRT	BLRT	Entropy	N	PP	Intercept (95% CI)	Slope linear (95% CI)
1	7907.945				617		10.1 (9.9, 10.4)	−0.27 (−0.42, −0.12)
2	7983.935	0.0001	<0.0001	0.8	125	0.895	13.5 (12.7, 14.3)	0.38 (−0.30, 1.07)
					492	0.955	9.2 (8.8, 9.5)	−0.48 (−0.64, −0.33)
3	7874.457	0.107	<0.0001	0.777	398	0.917	8.7 ( 8.2, 9.2)	−0.53 (−0.70, −0.37)
					30	0.844	14.1 (11.2, 17.0)	2.57 (0.35, 4.79)
					189	0.877	12.3 (11.5, 13.1)	−0.19 (−0.59, 0.21)
4	7804.208	0.1335	<0.0001	0.746	262	0.9	8.1 (7.7, 8.5)	−0.57 (−0.76, −0.38)
					253	0.794	11.0 (10.4, 11.5)	−0.50 (−0.78, −0.22)
					21	0.889	14.0 (10.3, 17.7)	3.20 (0.83, 5.56)
					81	0.836	13.1 (11.9, 14.4)	0.49 (−0.31, 1.28)

Abbreviations: BIC, Bayesian Information Criterion; BLRT, Bootstrap Likelihood Ratio Test; CI, confidence interval; LMR‐LRT, Vuong‐Lo–Mendell Rubin Likelihood Ratio Test; PP, posterior probabilities.

**FIGURE 1:**
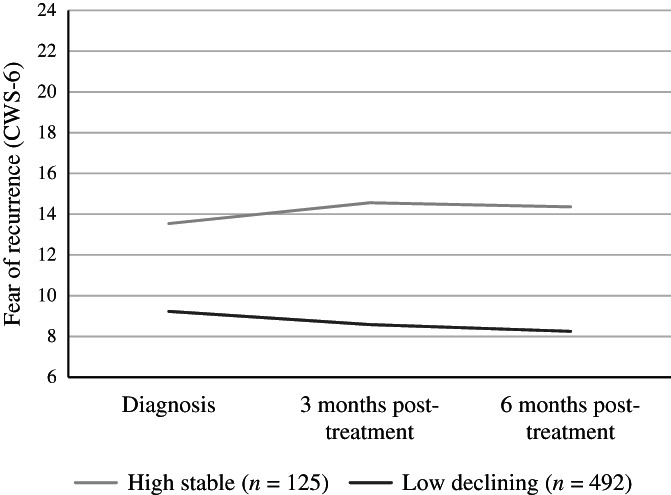
Trajectories of fear of cancer recurrence

The two subgroups differed in the baseline values (intercepts) of CWS‐6 scores (Figure 1). One subgroup consisted of 125 participants and was defined as “high stable,” as participants reported high baseline CWS‐6 scores (intercept 13.5 [95% CI 12.7, 14.3]) with a slope of 0.38 (−0.30, 1.07). The other subgroup consisted of 492 participants and was defined as “low declining,” as they reported low baseline CWS‐6 scores (intercept 9.2 [95% CI 8.8, 9.5]) with a slope of −0.48 (−0.64, −0.33).

### 3.4 Baseline factors associated with FCR trajectories

Comparisons of participant baseline characteristics between the two FCR trajectories are shown in Table 3. Younger persons (*F* = 23.97, *p* < 0.001) and females (chi‐square = 3.85, *p* = 0.05) were more likely to be in the “high stable” group. From the psychological factors, higher negative adjustment (*F* = 67.89, *p* < 0.001), more use of palliative coping (*F* = 17.30, *p* < 0.001), avoiding (*F* = 4.04, *p* = 0.05), seeking social support (*F* = 7.71, *p* < 0.01), passive coping (*F* = 72.37, *p* < 0.001), expressing emotions (*F* = 9.99, *p* < 0.01), and reassuring thoughts (*F* = 10.17, *p* < 0.01) and lower self‐efficacy (*F* = 10.42, *p* = 0.001) were significantly associated with high stable FCR.

**TABLE 3:** Analysis of Variance (ANOVA) and chi‐square test results for possible associated factors of fear of cancer recurrence trajectory membership (*N* = 617)

Factor	High Stable	Low Declining		
	*N* = 125	*N* = 492		
	Mean (SD)	Mean (SD)	*F*	*p*
Demographic				
Age	59.9 (9.7)	64.42 (9.1)	**23.97**	**< 0.001**
Psychological				
Summary positive adjustment^1^	49.8 (5.7)	50.2 (6.0)	0.41	0.52
Summary negative adjustment^2^	36.2 (6.4)	30.9 (6.1)	**67.89**	**< 0.001**
				
Active coping^3^	18.3 (3.9)	18.7 (3.7)	1.23	0.27
Palliative coping^4^	18.5 (3.6)	17.0 (3.5)	**17.3**	**< 0.001**
Avoiding^5^	15.8 (3.5)	15.1 (3.2)	**4.04**	**0.05**
Seeking social support^6^	13.6 (3.6)	12.7 (3.0)	**7.71**	**< 0.01**
Passive coping^7^	12.0 (3.1)	9.8 (2.4)	**72.37**	**< 0.001**
Expressing emotions^8^	5.2 (1.5)	4.7 (1.4)	**9.99**	**< 0.01**
Reassuring thoughts^9^	12.8 (2.5)	12.0 (2.5)	**10.17**	**< 0.01**
				
Self‐efficacy (GSE)^10^	31.4 (5.2)	33.0 (4.8)	**10.42**	**0.001**
	**No. of patients (%)**	**No. of patients (%)**	**Chi square**	***p***
Demographic				
Sex			**3.85**	**0.05**
Men	84 (67)	373 (76)		
Women	41 (33)	119 (24)		
				
Medical				
Cancer site^a^			2.84	0.42
Oral cavity	34 (29)	140 (29)		
Oropharynx	43 (37)	173 (36)		
Hypopharynx	11 (10)	28 (6)		
Larynx	28 (24)	141 (29)		
Clinical disease stage^b^			5.08	0.17
I	21 (17)	128 (26)		
II	27 (22)	85 (17)		
III	23 (18)	78 (16)		
IV	54 (43)	200 (41)		
HPV status^11^			0.66	0.42
Positive	20 (54)	92 (61)		
Negative	17 (46)	58 (39)		
Comorbidity^12^			0.62	0.1
None	39 (32)	145 (31)		
Mild	36 (30)	187 (40)		
Moderate	33 (27)	88 (19)		
Severe	14 (12)	47 (10)		

*Note*: Due to missing data: ^1^ *n* = 567, ^2^ *n* = 568, ^3^ *n* = 595, ^4^ *n* = 596, ^5^ *n* = 595, ^6^ *n* = 596, ^7^ *n* = 596, ^8^ *n* = 589, ^9^ *n* = 595, ^10^ *n* = 560, ^11^ *n* = 187, ^12^ *n* = 589.

Abbreviation: HPV, human papillomavirus.

^a^ Nineteen patients with unknown primary not included (*n* = 598).

^b^ One patient with cTNM Stage 0 not included (*n* = 616).

These factors were entered simultaneously in two binary logistic regression analyses with the low declining trajectory as reference group. There were no indications of multicollinearity. However, multiple factors were low or moderately correlated, indicating possible interdependence. The first model (Table 4) with age, sex, negative adjustment (MAC), and self‐efficacy was statistically significant (chi‐square = 94.53, *df* = 4, *p* < 0.001, Cox & Snell *R* ^2^ = 0.158, Nagelkerke = 0.248). Younger patients (OR = 0.93) and patients with more negative adjustment to cancer (OR = 1.17) were more likely to belong to the high stable than the low declining trajectory. The second model (Table 5) with age, sex, coping (UCL), and self‐efficacy was statistically significant (chi‐square = 89.48, *df* = 9, *p* < 0.001, Cox & Snell *R* ^2^ = 0.151, Nagelkerke = 0.236). Younger patients (OR = 0.95), patients who used more passive coping (*OR* = 1.34) and reassuring thoughts (OR = 1.16), and patients with lower self‐efficacy (OR = 0.94) were more likely to belong to the high stable than the low declining trajectory. Contrary to the univariate tests, patients with lower rather than higher avoidance were more likely to belong to the high stable trajectory (OR = 0.88).

**TABLE 4:** Binary logistic regression analysis results for possible associated factors of fear of cancer recurrence trajectory membership: age, sex, coping (MAC) and self‐efficacy (*n* = 551)

Factor	*B*	Wald	Exp (*B*) (95% CI)
Constant	−1.51	1.14	0.22
			
Age	−0.07	29.90**	0.93 (0.91, 0.96)
Sex	0.15	0.33	1.16 (0.70, 1.93)
			
Negative adjustment	0.15	45.42***	1.17 (1.11, 1.22)
			
Self‐efficacy	−0.02	0.77	0.98 (0.93, 1.03)

* *p* < 0.05, **p < 0.01, ***p < 0.001.

**TABLE 5:** Binary logistic regression analysis results for associated factors of fear of cancer recurrence trajectory membership: age, sex, coping (UCL) and self‐efficacy (*n* = 548)

Factor	*B*	Wald	Exp (*B*) (95% CI)
Constant	0.21	0.02	1.23
			
Age	−0.50	16.21***	0.95 (0.93, 0.97)
Sex	0.03	0.02	1.04 (0.61, 1.75)
			
Palliative coping	0.05	1.14	1.05 (0.96, 1.14)
Avoiding	−0.13	8.78**	0.88 (0.80, 0.96)
Seeking social support	−0.01	0.12	0.99 (0.91, 1.07)
Passive coping	0.29	32.08***	1.34 (1.21, 1.49)
Expressing emotions	0.00	0.00	1.00 (0.84, 1.19)
Reassuring thoughts	0.15	6.69*	1.16 (1.04, 1.30)
			
Self‐efficacy	−0.06	6.06*	0.94 (0.89, 0.99)

* *p* < 0.05, **p < 0.01, ***p < 0.001.

## 4 DISCUSSION

This study examined the course of FCR from diagnosis to 6 months post‐treatment in newly diagnosed HNC patients. At baseline, 30.8% of participants scored above the cut‐off threshold for high FCR, and 24.3% and 20.2% at 3 and 6 months post‐treatment, respectively. On group level, CWS‐6 scores declined significantly between diagnosis and 3 months post‐treatment and remained stable until 6 months post‐treatment. Two FCR subgroups were identified: “high stable” (20%) and “low declining” (80%). Patients with high stable FCR were younger, reported higher negative adjustment, passive coping and reassuring thoughts, less avoidant coping and lower self‐efficacy compared to the low declining group.

The proportion of patients scoring above the cut‐off for high FCR in our study is lower than reported in an earlier analysis of the NET‐QUBIC data (52.8%), and compared to previous studies in HNC (31%–83%).^7^, ^8^, ^9^, ^10^, ^11^, ^12^ Comparison of these percentages are limited by the variety of instruments and cut‐off scores used across studies. We used a conservatively chosen and validated cut‐off score, which was shown to be an accurate estimation of high FCR in HNC patients,^46^ while we in our previous study and others have used lower thresholds.

Declining FCR scores on group level after diagnosis have been reported in other populations^13^ and could reflect uncertainty and distress during the diagnostic and treatment period that partly decrease afterwards. However, both the effect size (*η* ^*2*^ = 0.029) and the difference in mean CWS‐6 scores between diagnosis and 3 months post‐treatment (0.56) in our study were relatively small, suggesting that the decline has limited clinical relevance. This is in line with a previous longitudinal study in HNC that did not find significant changes in FCR between pre‐treatment and 6–8 months post‐treatment^8^ and other longitudinal studies in which FCR was stable.^7^, ^9^, ^10^ With LCGA, we found a subgroup with high FCR, similar to what has been reported in trajectory studies in patients with breast cancer. One study found a low (75%) and high FCR (25%) group,^23^ and two other studies reported a moderate FCR group (47% and 40%) in addition to a low (40% and 38%) and high FCR group (13% and 22%).^22^, ^55^ Overall, these findings suggest that while most patients have low or moderate FCR, there is a distinct group of patients with persistent high FCR up to 6 months after treatment for whom psychological treatment may be indicated.

Consistent with previous studies, younger patients were more likely to report persistent high FCR.^9^, ^13^, ^16^, ^24^ They may experience the diagnosis as more unexpected^16^ and experience more consequences for important life domains including career and having children.^13^ FCR trajectory was not predicted by any medical variables which seem a robust finding across studies.^8^, ^9^, ^13^, ^16^, ^24^ Clinicians should be aware that FCR can be present regardless of disease severity and is rather related to patient‐related characteristics.

Little is known about coping strategies that might contribute to high FCR. In our study, high FCR was associated with coping strategies directed towards the fear rather than disengagement. High FCR was associated with negative adjustment to cancer, which is characterized by negative feelings (e.g., helplessness, anxiety, denial), and to less avoidance coping. Previous cross‐sectional and longitudinal studies, however, have reported that more avoidance coping was associated with high FCR, or found no significant association between avoidant coping and FCR.^29^, ^30^, ^31^, ^32^, ^33^, ^34^, ^56^ These different findings may be due to variation in the follow‐up time between studies. Higher FCR in our study was also related to greater use of reassuring thoughts (e.g., “worse things could happen”). Reassuring thoughts in this patient group might be characterized by repetitive thoughts rather than adaptive reappraisal of the situation.^33^ Consistent with previous studies, higher FCR was also related to passive coping, involving rumination, preoccupation with the situation and inability to act.^57^ Indeed, a recent qualitative study describes that patients with high FCR may use the same coping strategies as those with low FCR, but in a manner that is more time‐consuming or even obsessive.^58^ However, more in‐depth knowledge is necessary to clarify the underlying mechanisms.

The relation between FCR and self‐efficacy was inconclusive. In a similar study, patients with breast cancer with high FCR had lower self‐efficacy than those in the low FCR trajectory,^23^ while another longitudinal study showed no association.^38^ Thus, further research is needed to clarify whether and how self‐efficacy may play a role in FCR.

Our results might have implications for the ongoing international debate on the definition and measurement of “clinical FCR.” In a recent Delphi study,^18^ clinical FCR was characterized by high levels of preoccupation and worry that are persistent and hypervigilance to bodily symptoms. The existence of a distinct group with persistent high levels of FCR, as we found in our study, supports this definition, although it is important to realize that our results are limited to the first 6 months after treatment. The high and persistent FCR group also implicates the necessity of measuring FCR on multiple occasions before offering evidence‐based clinical interventions for FCR, as has been suggested in a recent study in patients with breast cancer.^22^ There is no consensus regarding coping strategies as a criterium for clinical FCR,^1^, ^18^ but our results suggest that dysfunctional coping might be characteristic of persisting high FCR, warranting further exploration.

Strengths of this study were a relatively large homogeneous multicenter sample of HNC patients followed immediately after diagnosis to 6 months post‐treatment, and the use of a statistical (bottom‐up) approach to identify subgroups of patients, enhancing the validity of the identified trajectories. The study also has some limitations that are important to consider. First, the NET‐QUBIC sample shows differences with the total HNC population (*N* = 9802) in the Netherlands Cancer Registry regarding sex (fewer females), age (on average 3 years younger), tumor site (less oral cavity and oropharynx cancer), and treatment modality.^43^ In our sample, CWS‐6 responders differed from non‐responders regarding tumor stage, HPV status and comorbidity and completers differed from non‐completers regarding HPV status and comorbidity, suggesting selection bias limiting generalizability. Second, the proportion of patients scoring above the cut‐off threshold for high FCR in this study was based on the CWS‐6 that has been validated against an established FCR questionnaire but not against a golden standard interview for FCR and therefore need to be interpreted carefully. Third, the length of follow‐up is limited to 6 months post‐treatment, and follow‐up moments are relatively close to each other. As a consequence, we were not able to assess whether the identified trajectories persist on the longer term. Therefore, we plan to replicate these analyses with longer follow‐up data, which will become available from the NET‐QUBIC study. Fourth, data for the time between the baseline measurement and end of treatment, which is variable across patients, were not available for the NET‐QUBIC cohort. We did report treatment type, which largely determines treatment duration. Finally, although the current study has a focus on associated psychological variables coping and self‐efficacy, other patient‐related factors have been associated with FCR both theoretically and empirically. Therefore, the unique role of coping and self‐efficacy must be interpreted with caution and future studies are warranted to test models with multiple factors over time in relation to FCR.

## 5 CONCLUSION

In a relatively large sample of patients with newly diagnosed HNC, the majority of patients had low declining FCR, but one in five patients experienced persistent high FCR from diagnosis up to 6 months post‐treatment. Younger age and coping strategies directed at the fear seemed important factors associated with high stable FCR. FCR interventions addressing these factors might be indicated for patients with high stable FCR.^59^

## CONFLICT OF INTEREST

The authors declare that there is no conflict of interest.

## Supporting information


**Table S1** Comparison between responders and non‐responders and between completers and non‐completers.

## Data Availability

Full dataset and statistical code is available via the NET‐QUBIC consortium (PI prof. dr I.M. Verdonck‐de Leeuw).

## References

[1] Lebel S , Ozakinci G , Humphris G , et al. From normal response to clinical problem: definition and clinical features of fear of cancer recurrence. Support Care Cancer. 2016;24(8):3265‐3268. 27169703 10.1007/s00520-016-3272-5

[2] Wells M , Cunningham M , Lang H , et al. Distress, concerns and unmet needs in survivors of head and neck cancer: a cross‐sectional survey. Eur J Cancer Care. 2015;24(5):748‐760. 10.1111/ecc.12370 26250705

[3] Kanatas A , Ghazali N , Lowe D , et al. Issues patients would like to discuss at their review consultation: variation by early and late stage oral, oropharyngeal and laryngeal subsites. Eur Arch Otorhinolaryngol. 2013;270(3):1067‐1074. 22743645 10.1007/s00405-012-2092-6

[4] Elaldi R , Roussel LM , Gal J , et al. Correlations between long‐term quality of life and patient needs and concerns following head and neck cancer treatment and the impact of psychological distress. A multicentric cross‐sectional study. Eur Arch Otorhinolaryngol. 2021;278(7):2437‐2445. 32901366 10.1007/s00405-020-06326-8

[5] Giuliani M , McQuestion M , Jones J , et al. Prevalence and nature of survivorship needs in patients with head and neck cancer. Head Neck. 2016;38(7):1097‐1103. 26894614 10.1002/hed.24411

[6] Henry M , Habib LA , Morrison M , et al. Head and neck cancer patients want us to support them psychologically in the posttreatment period: survey results. Palliat Support Care. 2014;12(6):481‐493. 24153040 10.1017/S1478951513000771

[7] Savard J , Ivers H . The evolution of fear of cancer recurrence during the cancer care trajectory and its relationship with cancer characteristics. J Psychosom Res. 2013;74(4):354‐360. 23497839 10.1016/j.jpsychores.2012.12.013

[8] Llewellyn CD , Weinman J , McGurk M , Humphris G . Can we predict which head and neck cancer survivors develop fears of recurrence? J Psychosom Res. 2008;65(6):525‐532. 19027440 10.1016/j.jpsychores.2008.03.014

[9] Ghazali N , Cadwallader E , Lowe D , Humphris G , Ozakinci G , Rogers SN . Fear of recurrence among head and neck cancer survivors: longitudinal trends. Psychooncology. 2013;22(4):807‐813. 22451036 10.1002/pon.3069

[10] Humphris GM , Rogers S , McNally D , Lee‐Jones C , Brown J , Vaughan D . Fear of recurrence and possible cases of anxiety and depression in orofacial cancer patients. Int J Oral Maxillofac Surg. 2003;32(5):486‐491. 14759106

[11] Campbell BH , Marbella A , Layde PM . Quality of life and recurrence concern in survivors of head and neck cancer. Laryngoscope. 2000;110(6):895‐906. 10852502 10.1097/00005537-200006000-00003

[12] Mirosevic S , Thewes B , van Herpen C , et al. Prevalence and clinical and psychological correlates of high fear of cancer recurrence in patients newly diagnosed with head and neck cancer. Head Neck. 2019;41(9):3187‐3200. 31173429 10.1002/hed.25812 PMC6771492

[13] Simard S , Thewes B , Humphris G , et al. Fear of cancer recurrence in adult cancer survivors: a systematic review of quantitative studies. J Cancer Survivorship. 2013;7(3):300‐322. 10.1007/s11764-013-0272-z 23475398

[14] Herschbach P , Dinkel A . Fear of progression. Recent Results Cancer Res. 2014;197:11‐29. 24305766 10.1007/978-3-642-40187-9_2

[15] Lee‐Jones C , Humphris G , Dixon R , Hatcher MB . Fear of cancer recurrence‐‐a literature review and proposed cognitive formulation to explain exacerbation of recurrence fears. Psychooncology. 1997;6(2):95‐105. 9205967 10.1002/(SICI)1099-1611(199706)6:2<95::AID-PON250>3.0.CO;2-B

[16] Crist JV , Grunfeld EA . Factors reported to influence fear of recurrence in cancer patients: a systematic review. Psychooncology. 2013;22(5):978‐986. 22674873 10.1002/pon.3114

[17] Williams JTW , Pearce A , Smith A . A systematic review of fear of cancer recurrence related healthcare use and intervention cost‐effectiveness. Psychooncology. 2021;30(8):1185‐1195. 33880822 10.1002/pon.5673

[18] Mutsaers B , Butow P , Dinkel A , et al. Identifying the key characteristics of clinical fear of cancer recurrence: an international Delphi study. Psychooncology. 2020;29(2):430‐436. 31713279 10.1002/pon.5283

[19] Henselmans I , Helgeson VS , Seltman H , de Vries J , Sanderman R , Ranchor AV . Identification and prediction of distress trajectories in the first year after a breast cancer diagnosis. Health Psychol. 2010;29(2):160‐168. 20230089 10.1037/a0017806

[20] Zhu L , Ranchor AV , Helgeson VS , et al. Benefit finding trajectories in cancer patients receiving psychological care: predictors and relations to depressive and anxiety symptoms. Br J Health Psychol. 2018;23(2):238‐252. 29139593 10.1111/bjhp.12283

[21] Dunn J , Ng SK , Holland J , et al. Trajectories of psychological distress after colorectal cancer. Psychooncology. 2013;22(8):1759‐1765. 23125004 10.1002/pon.3210

[22] Custers JA , Kwakkenbos L , van der Graaf WT , Prins JB , Gielissen MF , Thewes B . Not as stable as we think: a descriptive study of 12 monthly assessments of fear of cancer recurrence among curatively‐treated breast cancer survivors 0–5 years after surgery. Front Psychol. 2020;11:580979. 33224072 10.3389/fpsyg.2020.580979 PMC7667242

[23] McGinty HL , Small BJ , Laronga C , Jacobsen PB . Predictors and patterns of fear of cancer recurrence in breast cancer survivors. Health Psychol. 2016;35(1):1‐9. 26030308 10.1037/hea0000238

[24] Koch L , Jansen L , Brenner H , Arndt V . Fear of recurrence and disease progression in long‐term (≥5 years) cancer survivors‐‐a systematic review of quantitative studies. Psychooncology. 2013;22(1):1‐11. 10.1002/pon.3022 22232030

[25] Pang C , Humphris G . The relationship between fears of cancer recurrence and patient gender: a systematic review and meta‐analysis. Front Psychol. 2021;12:640866. 33692731 10.3389/fpsyg.2021.640866 PMC7937637

[26] Fardell JE , Thewes B , Turner J , et al. Fear of cancer recurrence: a theoretical review and novel cognitive processing formulation. J Cancer Surviv. 2016;10(4):663‐673. 26782171 10.1007/s11764-015-0512-5

[27] Lazarus RS , Folkman S . Stress, Appraisal, and Coping. Springer Pub. Co.; 1984.

[28] Custers JA , Gielissen MF , de Wilt JH , et al. Towards an evidence‐based model of fear of cancer recurrence for breast cancer survivors. J Cancer Surviv. 2017;11(1):41‐47. 27412726 10.1007/s11764-016-0558-z PMC5266772

[29] Wade TD , Nehmy T , Koczwara B . Predicting worries about health after breast cancer surgery. Psychooncology. 2005;14(6):503‐509. 15390218 10.1002/pon.866

[30] Stanton AL , Danoff‐Burg S , Huggins ME . The first year after breast cancer diagnosis: hope and coping strategies as predictors of adjustment. Psychooncology. 2002;11(2):93‐102. 11921325 10.1002/pon.574

[31] Hilton BA . The relationship of uncertainty, control, commitment, and threat of recurrence to coping strategies used by women diagnosed with breast cancer. J Behav Med. 1989;12(1):39‐54. 2746642 10.1007/BF00844748

[32] Mehnert A , Berg P , Henrich G , Herschbach P . Fear of cancer progression and cancer‐related intrusive cognitions in breast cancer survivors. Psychooncology. 2009;18(12):1273‐1280. 19267364 10.1002/pon.1481

[33] Simard S , Savard J , Ivers H . Fear of cancer recurrence: specific profiles and nature of intrusive thoughts. J Cancer Surviv. 2010;4(4):361‐371. 20617394 10.1007/s11764-010-0136-8

[34] Skaali T , Fossa SD , Bremnes R , et al. Fear of recurrence in long‐term testicular cancer survivors. Psychooncology. 2009;18(6):580‐588. 18855944 10.1002/pon.1437

[35] Bandura A . Human agency in social cognitive theory. Am Psychol. 1989;44(9):1175‐1184. 2782727 10.1037/0003-066x.44.9.1175

[36] Leventhal H , Halm E , Horowitz C , Leventhal E , Ozakinci G . Living with chronic illness: a contextualised, self‐regulation approach. In: Sutton S , Baum A , Johnston M , eds. The Sage Handbook of Health Psychology. SAGE; 2008:197‐240.

[37] Galica J , Maheu C , Brennenstuhl S , Townsley C , Metcalfe K . Examining predictors of fear of cancer recurrence using Leventhal's commonsense model: distinct implications for oncology nurses. Cancer Nurs. 2021;44(1):3‐12. 31868820 10.1097/NCC.0000000000000760

[38] Melchior H , Buscher C , Thorenz A , Grochocka A , Koch U , Watzke B . Self‐efficacy and fear of cancer progression during the year following diagnosis of breast cancer. Psychooncology. 2013;22(1):39‐45. 21898655 10.1002/pon.2054

[39] Torbit LA , Albiani JJ , Crangle CJ , Latini DM , Hart TL . Fear of recurrence: the importance of self‐efficacy and satisfaction with care in gay men with prostate cancer. Psychooncology. 2015;24(6):691‐698. 25060033 10.1002/pon.3630 PMC4416068

[40] Ziner KW , Sledge GW , Bell CJ , Johns S , Miller KD , Champion VL . Predicting fear of breast cancer recurrence and self‐efficacy in survivors by age at diagnosis. Oncol Nurs Forum. 2012;39(3):287‐295. 22543387 10.1188/12.ONF.287-295 PMC5018900

[41] Hall DL , Luberto CM , Philpotts LL , Song R , Park ER , Yeh GY . Mind‐body interventions for fear of cancer recurrence: a systematic review and meta‐analysis. Psychooncology. 2018;27(11):2546‐2558. 29744965 10.1002/pon.4757 PMC6488231

[42] Tauber NM , O'Toole MS , Dinkel A , et al. Effect of psychological intervention on fear of cancer recurrence: a systematic review and meta‐analysis. J Clin Oncol. 2019;37(31):2899‐2915. 31532725 10.1200/JCO.19.00572 PMC6823887

[43] Verdonck‐de Leeuw IM , Jansen F , Brakenhoff RH , et al. Advancing interdisciplinary research in head and neck cancer through a multicenter longitudinal prospective cohort study: The Netherlands QUality of life and BIomedical Cohort (NET‐QUBIC) data warehouse and biobank. BMC Cancer. 2019;19(1):765. 31382921 10.1186/s12885-019-5866-z PMC6683500

[44] van Nieuwenhuizen AJ , Buffart LM , Smit JH , et al. A comprehensive assessment protocol including patient reported outcomes, physical tests, and biological sampling in newly diagnosed patients with head and neck cancer: is it feasible? Support Care Cancer. 2014;22(12):3321‐3330. 25110298 10.1007/s00520-014-2359-0 PMC4218976

[45] Lerman C , Daly M , Masny A , Balshem A . Attitudes about genetic testing for breast‐ovarian cancer susceptibility. J Clin Oncol. 1994;12(4):843‐850. 8151327 10.1200/JCO.1994.12.4.843

[46] Custers JAE , Kwakkenbos L , van de Wal M , Prins JB , Thewes B . Re‐validation and screening capacity of the 6‐item version of the Cancer Worry Scale. Psychooncology. 2018;27(11):2609‐2615. 29843189 10.1002/pon.4782

[47] Watson M , Homewood J . Mental adjustment to cancer scale: psychometric properties in a large cancer cohort. Psychooncology. 2008;17(11):1146‐1151. 18626853 10.1002/pon.1345

[48] Watson M , Greer S , Young J , Inayat Q , Burgess C , Robertson B . Development of a questionnaire measure of adjustment to cancer: the MAC scale. Psychol Med. 1988;18(1):203‐209. 3363039 10.1017/s0033291700002026

[49] Braeken AP , Kempen GI , Watson M , Houben RM , Gils FC , Lechner L . Psychometric properties of the Dutch version of the Mental Adjustment to Cancer scale in Dutch cancer patients. Psychooncology. 2010;19(7):742‐749. 19824025 10.1002/pon.1628

[50] Schreurs P , Tellegen B , Willige G . Gezondheid, stress en coping: de ontwikkeling van de Utrechtse coping‐lijst [Health, stress and coping: The development of the Utrecht Coping List]. Gedrag. 1984;12:101‐117.

[51] Schreurs PJG , van de Willige G , Brosschot JF , Tellegen B , GMH G . De utrechtse coping lijst omgaan met problemen en gebeurtenissen. Pearson Assessment & Information B.V; 1993.

[52] Schwartzer R , Jerusalem M . Generalized self‐efficacy scale. In: Weinman J , Wright S , Johnston M , eds. Measures in Health Psychology: a user's Portfolio. Nfer‐Nelson; 1995:35‐37.

[53] Paleri V , Wight RG . Applicability of the adult comorbidity evaluation ‐ 27 and the Charlson indexes to assess comorbidity by notes extraction in a cohort of United Kingdom patients with head and neck cancer: a retrospective study. J Laryngol Otol. 2002;116(3):200‐205. 11893262 10.1258/0022215021910528

[54] Jung T , Wickrama KAS . An introduction to latent class growth analysis and growth mixture modeling. Soc Personal Psychol Compass. 2008;2(1):302‐317.

[55] Shim EJ , Jeong D , Lee SB , Min YH . Trajectory of fear of cancer recurrence and beliefs and rates of medication adherence in patients with breast cancer. Psychooncology. 2020;29(11):1835‐1841. 32720375 10.1002/pon.5497

[56] Aarstad AK , Aarstad HJ , Bru E , Olofsson J . Psychological coping style versus disease extent, tumour treatment and quality of life in successfully treated head and neck squamous cell carcinoma patients. Clin Otolaryngol. 2005;30(6):530‐538. 16402979 10.1111/j.1749-4486.2005.01114.x

[57] Johansson M , Ryden A , Finizia C . Mental adjustment to cancer and its relation to anxiety, depression, HRQL and survival in patients with laryngeal cancer ‐ a longitudinal study. BMC Cancer. 2011;11:283. 21718478 10.1186/1471-2407-11-283 PMC3136424

[58] Thewes B , Lebel S , Seguin Leclair C , Butow P . A qualitative exploration of fear of cancer recurrence (FCR) amongst Australian and Canadian breast cancer survivors. Support Care Cancer. 2016;24(5):2269‐2276. 26581900 10.1007/s00520-015-3025-x PMC4805701

[59] van de Wal MA , Gielissen MF , Servaes P , Knoop H , Speckens AE , Prins JB . Study protocol of the SWORD‐study: a randomised controlled trial comparing combined online and face‐to‐face cognitive behaviour therapy versus treatment as usual in managing fear of cancer recurrence. BMC Psychol. 2015;3(1):12. 25977758 10.1186/s40359-015-0068-1 PMC4431367

