# Supplementary material for: Prospective longitudinal study on fear of cancer recurrence in patients newly diagnosed with head and neck cancer: Course, trajectories, and associated factors
Source: Head Neck. 2022 Jan 27;44(4):914–25. doi: 10.1002/hed.26985 (PMC9305148; doi:10.1002/hed.26985)
Supplement: Supplementary file 1 — Table S1 Comparison between responders and non‐responders and between completers and non‐completers. [file HED-44-914-s001.docx]

**TABLE S1.** Comparison between responders and non-responders and between completers and non-completers.

| **Factor** | **Responders**  **N=617** | **Non-responders**  **N=122** |  |  | **Completers**  **N=417** | **Non-completers**  **N=200** |  |  |
| --- | --- | --- | --- | --- | --- | --- | --- | --- |
|  | **Mean (SD)** | **Mean (SD)** | ***F*** | ***p*** | **Mean (SD)** | **Mean (SD)** | ***F*** | ***p*** |
| Age | 63.5 (9.4) | 62.0 (11.1) | 6.37 | .11 | 64.0 | 62.5 | .16 | .69 |
|  | **No. of**  **patients (%)** | **No. of**  **patients (%)** | **X^2^** | ***p*** | **No. of**  **patients (%)** | **No. of**  **patients (%)** | **X^2^** | ***p*** |
| Sex  Men  Women  Cancer site^*^  Oral cavity  Oropharynx  Hypopharynx  Larynx  Clinical disease stage^†^  I  II  III  IV  Treatment  Surgery  Radiotherapy  Chemotherapy  CO2-laser^‡^  HPV status^1^  Positive  Negative  Comorbidity^2^  None  Mild  Moderate  Severe | 457 (74)  160 (26)  174 (29)  216 (36)  39 (7)  169 (28)  149 (24)  112 (18)  101 (16)  254 (41)  202 (33)  485 (79)  190 (30.8)  46 (13.4)  112 (60)  75 (40)  184 (31)  223 (38)  121 (21)  61 (10) | 92 (75)  30 (25)  25 (21)  46 (38)  13 (11)  36 (30)  13 (11)  20 (16)  26 (21)  63 (52)  29 (24)  101 (83)  48 (39)  5 (8.2)  18 (43)  24 (57)  20 (18)  41 (37)  34 (31)  15 (14) | .10  5.27  **12.67**  3.81  1.08  3.4  1.28  **4.06**  **10.83** | .76  .15  **< .01**  .05  .30  .07  .26  **.04**  **.01** | 310 (74)  107 (26)  118 (29)  143 (36)  25 (6)  115 (29)  105 (25)  80 (19)  66 (16)  165 (40)  136 (33)  321 (77)  122 (29)  31 (13.3)  80 (66)  41 (34)  138 (34)  163 (41)  68 (17)  32 (8) | 147 (74)  53 (27)  56 (28)  73 (37)  14 (7)  54 (27)  44 (22)  32 (16)  35 (18)  89 (45)  66 (33)  164 (82)  68 (34)  15 (13.6)  32 (49)  34 (52)  46 (25)  60 (32)  53 (28)  29 (15) | .05  .35  2.3  .01  2.03  1.43  .01  **5.53**  **21.35** | .82  .95  .50  .92  .16  .23  .93  **.02**  **<.001** |

*Notes:* Due to missing data: ^1^ responders *n*=187; non-responders *n*=42; completers *n*=121; non-completers *n*=66; ^2^ responders *n*=589; non-responders *n*=110; completers *n*=401; non-completers *n*=188

Comparison: Age was tested with an independent samples t-test, the distributions of the other variables were tested with chi-squared tests.

^*^ patients with unknown primary not included (responders *n*=598; non-responders *n*=120; completers *n*=401; non-completers *n*=197)

^†^ one patient with cTNM stage 0 not included (responders *n*=616; completers *n*=416).

^‡^ applicable only in oral cavity & larynx cancer (responders *n*=343; non-responders *n*=61; completers *n*=233; non-completers *n*=110)
